# Selenium Biofortification of Soybean Sprouts: Effects of Selenium Enrichment on Proteins, Protein Structure, and Functional Properties

**DOI:** 10.3389/fnut.2022.849928

**Published:** 2022-05-03

**Authors:** Yatao Huang, Bei Fan, Ningyu Lei, Yangyang Xiong, Yanfang Liu, Litao Tong, Fengzhong Wang, Philippe Maesen, Christophe Blecker

**Affiliations:** ^1^Key Laboratory of Agro-Products Quality and Safety Control in Storage and Transport Process, Ministry of Agriculture and Rural Affairs, Institute of Food Science and Technology, Chinese Academy of Agricultural Sciences, Beijing, China; ^2^Department of Food Science and Formulation, Bureau d'études Environnement et Analyses (BEAGx), Gembloux Agro-Bio Tech, Université de Liège, Gembloux, Belgium

**Keywords:** selenium biofortification, germinated soybean, protein structure, functional properties, antioxidant capacity

## Abstract

Selenium (Se) biofortification during germination is an efficient method for producing Se-enriched soybean sprouts; however, few studies have investigated Se distribution in different germinated soybean proteins and its effects on protein fractions. Herein, we examined Se distribution and speciation in the dominant proteins 7S and 11S of raw soybean (RS), germinated soybean (GS), and germinated soybean with Se biofortification (GS-Se). The effects of germination and Se treatment on protein structure, functional properties, and antioxidant capacity were also determined. The Se concentration in GS-Se was 79.8-fold higher than that in GS. Selenomethionine and methylselenocysteine were the dominant Se species in GS-Se, accounting for 41.5–80.5 and 19.5–21.2% of the total Se with different concentrations of Se treatment, respectively. Se treatment had no significant effects on amino acids but decreased methionine in 11S. In addition, the α-helix contents decreased as the Se concentration increased; the other structures showed no significant changes. The Se treatment also had no significant effects on the water and oil-holding capacities in protein but increased the foaming capacity and emulsion activity index (EAI) of 7S, but only the EAI of 11S. The Se treatment also significantly increased the antioxidant capacity in 7S but not in 11S. This study indicates that the dominant proteins 7S and 11S have different Se enrichment abilities, and the protein structures, functional properties, and antioxidant capacity of GS can be altered by Se biofortification.

## Introduction

Soybean is a major source of high-quality plant protein, providing essential amino acids and proteins with a variety of functional properties at low cost (1, 2). Soybeans also have multiple roles in food processing due to their unique protein-related food texture (3), high water-holding capacities, and foaming properties. Thus, the soybean has been widely used in the processing of sausages (4), beverages (5), bread, and cakes (6) to modify the food texture. The structure and functionality of soybean proteins are also important for the nutrition and quality of food products (7).

Selenium (Se) is an important micronutrient for both animal and human health, and Se intake should meet the recommended adult dietary allowance of 50–60 μg/day (8). However, the Se contents in agricultural soils are considerably low in some regions, such as China, New Zealand, and parts of Europe, where insufficient Se intake from plant-derived food has become a public health issue because of low Se levels in the environment (9). Selenoproteins play important roles in both human and animal health. For example, glutathione peroxidase (GPx) can protect the human body from oxidative stress (10). Indeed, the antioxidant activity of Se has attracted increasing research interest (11, 12), and the enrichment of foods through Se biofortification has become a popular strategy to promote adequate dietary intake of Se.

Excessive Se intake can result in Se toxicity, which largely depends on both the concentration of total Se and the Se speciation in dietary materials. Different Se species are associated with different metabolism pathways and further result in different levels of Se mobility, bioavailability, and toxicity (13, 14). In general, selenite (${\text{SeO}}_{3}^{2-}$) is known to be more toxic to organisms than selenate and other organic Se compounds, such as selenomethionine (SeMet), selenocystine (SeCys_2_), and methylselenocysteine (MeSeCys). Previous studies also found that these five Se compounds are the dominant chemical compounds of Se accumulated in Se-enriched soybean (7). Organic Se compounds have relatively low toxicity and high nutritional value compared with inorganic Se compounds (15), and those seleno-amino acids are incorporated into proteins, namely selenoproteins. Chan et al. found that over 80% of the total Se is bonded to high-molecular-weight proteins in Se-enriched soybean (16). Se-containing proteins in food products are ideal dietary sources of Se intake and are used as food supplement products *via* appropriate processing procedures in the agri-food industry (17).

However, Se is distributed unequally in protein fractions. Soybean proteins mostly consist of globulins, and four kinds, namely, 2S, 7S, 11S, and 15S, account for 15, 34, 41.9, and 9.1% of all soybean globulins, respectively. 7S and 11S account for 75.9% of the total globulins (18), as the dominant proteins, they have been the focus in regards to soybean and protein processing in previous studies (19, 20). A previous study found that the abilities of different protein fractions to enrich Se differ from each other: the concentration of Se in 11S was 38% higher than that in 7S in soybeans cultivated by Se foliar spray, and 11S had a higher Se enrichment ability than 7S (7). However, there is less information on the distribution and species of Se in Se biofortification-germinated soybean proteins, and the Se distribution and these species should be clarified.

To better utilize Se-enriched germinated soybean protein, the structure of the protein should be known. Previous studies have indicated that protein structure, functional properties, and antioxidant capacity are changed during germination (21–23). Simultaneously, Se can bind to soybean proteins by S–S, Se–S, and Se–Se bonds; therefore, parts of the secondary structure, such as the α-helix, β-sheet, and random coil, are influenced (24). A previous study found that Se could influence the secondary structures of 7S and 11S in raw soybeans (25), but Deng et al. found that there was no significant effect on protein secondary structure in soybeans enriched through Se foliar spraying (7). The influence of Se on the secondary structure of raw soybean protein has not yet been clarified, and to the best of our knowledge, Se-enriched germinated soybean proteins have not been studied.

Changes in protein components and structure can affect their functional properties and antioxidant capacity. The functionality of soybean proteins, including their water-holding capacity (WHC), oil-holding capacity (OHC), foaming capacity (FC), and emulsion activity index (EAI), will influence how soybean proteins work in food processing. Germination can cause the breakdown of macro-molecular proteins, and the secondary structure of the protein changes after germination, affecting both the FC and EAI of proteins (26). Previous studies have focused on the functionality of Se-enriched soybean (7, 25), but less is known about germinated Se biofortification soybeans. Previous studies investigated the antioxidant capacity of germinated soybean (22, 27) and found that germination could improve the antioxidant capacity. The antioxidant capacity of Se-containing protein from *Ganoderma lucidum* was three times higher than that of the proteins without Se treatment. In addition, the capacity was correlated with the Se content in protein (28). However, the effect of Se biofortification on soybean sprout proteins remains unknown.

As the application of Se biofortification during germination in the food industry has increased, Se distribution and its effects on protein structure and functionality need to be clarified. Previous studies have confirmed that Se biofortification can promote the content of Se and influence the protein structure and functional ability of Se-enriched soybeans; however, its effects on germinated soybean proteins remain unclear. In addition, few studies have assessed Se distribution and speciation in biofortified germinated soybean proteins and their effects on the structure of different soybean protein fractions. Therefore, the purpose of our study was to investigate the effects of Se on the structure, functional properties, and antioxidant capacity of germinated soybean proteins.

## Materials and Methods

### Selenium-Enriched Soybean Sprouts

Soybean (Zhonghuang 13) seeds were provided by the Institute of Crop Science of CAAS (Beijing, China). The soybean seeds were surface-sterilized with 0.1% NaClO solution for 5 min, then washed five times with deionised water (Milli-RO Plus; MilliporeSigma, Burlington, MA, USA), and soaked in different concentrations of Se solution (0, 5, 30, and 60 mg/L of sodium selenite solution) at a ratio of 1:5 (w/v) for 6 h. Later, the seeds were placed in plant tissue culture containers with two layers of gauze and then placed in an incubator in the dark with a controlled temperature of 25°C. The seeds were sprayed with deionised water (~350 ml) for 5 s every 10 h during germination, and soybean sprouts were harvested every 24 h until 120 h. Raw soybean (RS), germinated soybean in the control (GS), and germinated soybean under Se treatment (GS-Se5/30/60) were freeze-dried and ground with a grinder to pass through a 40 mesh sieve. The powders were sealed in bags until use. 7S and 11S were prepared according to the previous methods (7, 29), which are presented in the Supplementary Material.

### Total and Species Se Analysis

Approximately 0.5 g of soybean sprout powder was mixed with 6 ml of HNO_3_ and 2 ml of H_2_O_2_ (30%) in 50-ml polypropylene tubes. The mixture was digested at 120°C until white fumes appeared. Concentrated HCl (5 ml) was added as a reductant to reduce selenate to selenite. The solution was diluted with deionised water to 25 ml, and Se was detected using hydride generation atomic fluorescence spectrometry (HG-AFS 9230; Beijing Titan Instruments, Beijing, China). The limits of quantification and detection for Se based on plant dry weight for the entire procedure were estimated to be 0.48 and 0.36 μg/kg, respectively.

Samples were prepared according to a previous study (30), with some modifications. A powdered sample (0.1 g) was transferred into a 15-ml plastic tube, and 10 ml of Tris-HCl (75 mmol/L, pH 7.5) and 10 mg protease XIV (Sigma-Aldrich, St. Louis, MO, USA) were added. The mixture was homogenized using ultrasound at 37°C for 18 h. The supernatant was collected after being centrifuged, and the filtrate was obtained by a 0.22-μm hydrophilic filter and stored at 4°C until Se speciation analysis.

Instrumental analysis was conducted according to a previous study (31), with some modifications. The Se species were determined using a high-performance liquid chromatography (HPLC) system (U3000; Thermo Fisher Scientific, Waltham, MA, USA) equipped with a ZORBAX SB-Aq column (4.6 × 250 mm, particle size, 5 μm; Thermo Fisher Scientific). The flow rate of mobile phase (10 mM citric acid, 0.5 mM sodium 1-hexanesulfonate, 2% methanol, pH 5.5) was 0.8 ml/min. The outlet of the HPLC system was coupled to an ICP-MS instrument (X Series 2; Thermo Fisher Scientific; USA). The column outlet of the HPLC system was connected to a Micro-mist nebuliser using PEEK tubing (0.25 mm i.d. × 10^4^ cm length).

### Amino Acids and Protein Subunit Analysis

Amino acids in samples were determined as previously reported (7) and are presented in the Supplementary Material. The protein subunit was analyzed by sodium dodecyl sulfate-polyacrylamide gel electrophoresis (SDS-PAGE) according to a previous study (26), with minor modifications. Then, 2 mg/ml soybean protein sample solution was first prepared, and the buffer was 12% separating gel and stacking gel. After being stained, gels were decolorised until the background was clear. The standard marker was a 10–1,000 kDa molecular weight protein.

### Fourier Transform Infrared Spectroscopy Analysis

Fourier transform infrared (FTIR) spectroscopy analysis of protein was performed according to a previous study (30). Approximately 1 mg dried sample was mixed with 100 mg KBr and pressed into a pellet. The pellet was analyzed by a Nicolet 5700 FTIR spectrometer (400–4,000 cm^−1^ wavenumber) with a 4 cm^−1^ resolution and an accumulation of 32 scans. Data were acquired and processed using Omnic 8.0 software (Thermo Fisher Scientific Inc., Madison, WI, USA) and Peakfit 4.12 (Systat Software, San Jose, CA, USA).

### Water and Oil-Holding Capacity Analysis

The WHC and OHC were determined according to a previous report (7). About 0.5 g of sample (W_0_) was placed into a centrifuge tube (25 ml) and then weighed (W_1_). Then, 10 ml water or oil was added, and the sample was allowed to sit for 30 min before centrifuging for 10 min (6,000 × *g*). The samples together with the centrifuge tube were weighed after removing the upper layer of water or oil (W_2_). WHC and OHC were calculated using the following equation:


(1) $$WHC\;(OHC)\;=\frac{W_{2}-W_{1}}{W_{0}}$$

### Foaming Capacity and Foam Stability Analysis

FC and FS were assessed according to a previous report (32). A 30 ml sample of 1% (w/v) protein solution (pH 7.0) was homogenized in a mechanical homogeniser at 13,000 rpm for 3 min. FC was calculated using the following equation:


(2) $$FC\;(\%\;)=\frac{V_{1}-V_{0}}{V_{0}}$$

(3) $$FS\;(\%\;)=\frac{V_{2}-V_{0}}{V_{1}}$$

where V_0_ and V_1_ are the volumes before and after whipping, respectively, and V_2_ is the volume after standing for 30 min.

### Emulsifying Activity Index and Emulsifying Stability Index Analysis

EAI and ESI were assessed according to a previous report (33) and calculated using Equations 4, 5. Aqueous emulsifier solution (9 ml, containing 1% protein) and 3 ml soybean oil were added into a tube and blended. Then, 20 μl of the emulsion was pipetted into 5 ml of 0.1% sodium dodecyl sulfate aqueous solution. Then, the absorbance was read at 500 nm at 0 (A_0_) and 30 (A_30_) min.


(4) $$EAI\;(m2/g)=\frac{2(T\;\times \;A_{0}\;\times \;N)}{C\;\times \;\Phi \;\times \;10000}$$

(5) $$ESI\;(min)\;=\;\frac{A_{0}\;\times \;t}{A_{0}-\;A_{30}}$$

where T is 2.303, A_0_ and A_30_ are the absorbances at 0 and 30 min, respectively, N is 250, C is the initial protein concentration, and Φ is the volume fraction of the emulsion (0.2).

### Antioxidant Activity Analysis

The 1,1-diphenyl-2-picrylhydrazyl (DPPH) radical-scavenging ability of 7S and 11S were analyzed according to a previous study (34). Briefly, 2.0 ml of a water solution of the samples at 1.0, 2.0, 3.0, 4.0, and 5.0 mg/ml was mixed with 2 ml alcoholic solution of DPPH (1.0 × 10^−4^ M) and then measured after the reaction. The DPPH radical-scavenging ability was calculated using the following equation:


(6) $$\begin{array}{l} DPPH\;radical\;scavenging\;activity\;(\%)\\ \;=\;\left[1-\frac{A_{2}-A_{1}}{A_{0}}\right]\;\times \;100\% \end{array}$$

where A_0_ is the absorbance of the deionised water; A_1_ and A_2_ are the absorbances of the sample with ethanol solution and DPPH, respectively.

The •OH radical-scavenging ability of 7S and 11S was analyzed according to a previous study (35). Briefly, 2.0 ml of a water solution of the samples at 1.0, 2.0, 3.0, 4.0, and 5.0 mg/ml were mixed with 1.0 ml of 9.0 mmol/L salicylic acid ethanol solution and 1.0 ml of 9.0 mmol/L FeSO_4_ and H_2_O_2_ aqueous solution and then measured after the reaction. The •OH radical-scavenging ability was calculated by the following equation:


(7) $$\begin{array}{l} OH\;radical\;scavenging\;ability\;(\%)\\ =\;1-\frac{A_{2}-A_{1}}{A_{0}}\;\times \;100\% \end{array}$$

where A_0_ is the absorbance of deionised water, and A_1_ and A_2_ are the absorbances of the sample with deionised water and H_2_O_2_, respectively.

### Data Analysis

Analysis of variance was performed followed by Duncan's test (*p* < 0.05) in the SPSS 19 software (IBM Corp., Armonk, NY, USA). For each tested group, the sample sizes in “amino acid concentration” are 2, and the other sample sizes are 3, respectively. Figures were drawn by Origin 2018 (OriginLab Inc., Northampton, MA, USA).

## Results

### Total Se in Soybean and Its Proteins

The total Se of soybean/sprout powder (SP), 7S, and 11S in RS, GS, and GS-Se with different concentrations of Se treatment is summarized in Table 1. There was an increase in total Se content, as the Se concentration in the solution increased from 5 to 60 mg/L; the total Se in SP, 7S, and 11S of GS-Se60 was 2,882, 2,035, and 4,301 μg/kg, and elevated by 79.8, 60.2, and 73.9 times, respectively, compared with the control group. The total Se in 11S was significantly higher than that in 7S by 69.6, 72.1, and 111% in RS, GS, and GS-Se30, respectively.

**Table 1:** Total selenium (Se) concentration in raw soybean (RS), germinated soybean (GS), and germinated soybean with Se biofortification (GS-Se) with different Se treatment concentrations (μg/kg).

**Samples**	**Soybean/sprouts**	**7S**	**11S**
RS	33.03 ± 2.44 b	30.41 ± 1.26 b	51.59 ± 2.23 a
GS	36.12 ± 2.57 b	33.82 ± 0.98 b	58.21 ± 4.40 a
GS-Se5	242.5 ± 12.74 b	227.3 ± 14.66 b	357.0 ± 22.10 a
GS-Se30	1,297 ± 35.53 b	952.1 ± 37.34 c	2,040 ± 40.98 a
GS-Se60	2,882 ± 46.01 b	2,035 ± 90.51 c	4,301 ± 214.5 a

*Different letters are used to show significant differences at various treatments (p < 0.05)*.

### Se Species in Different Proteins

**Figure 1:**
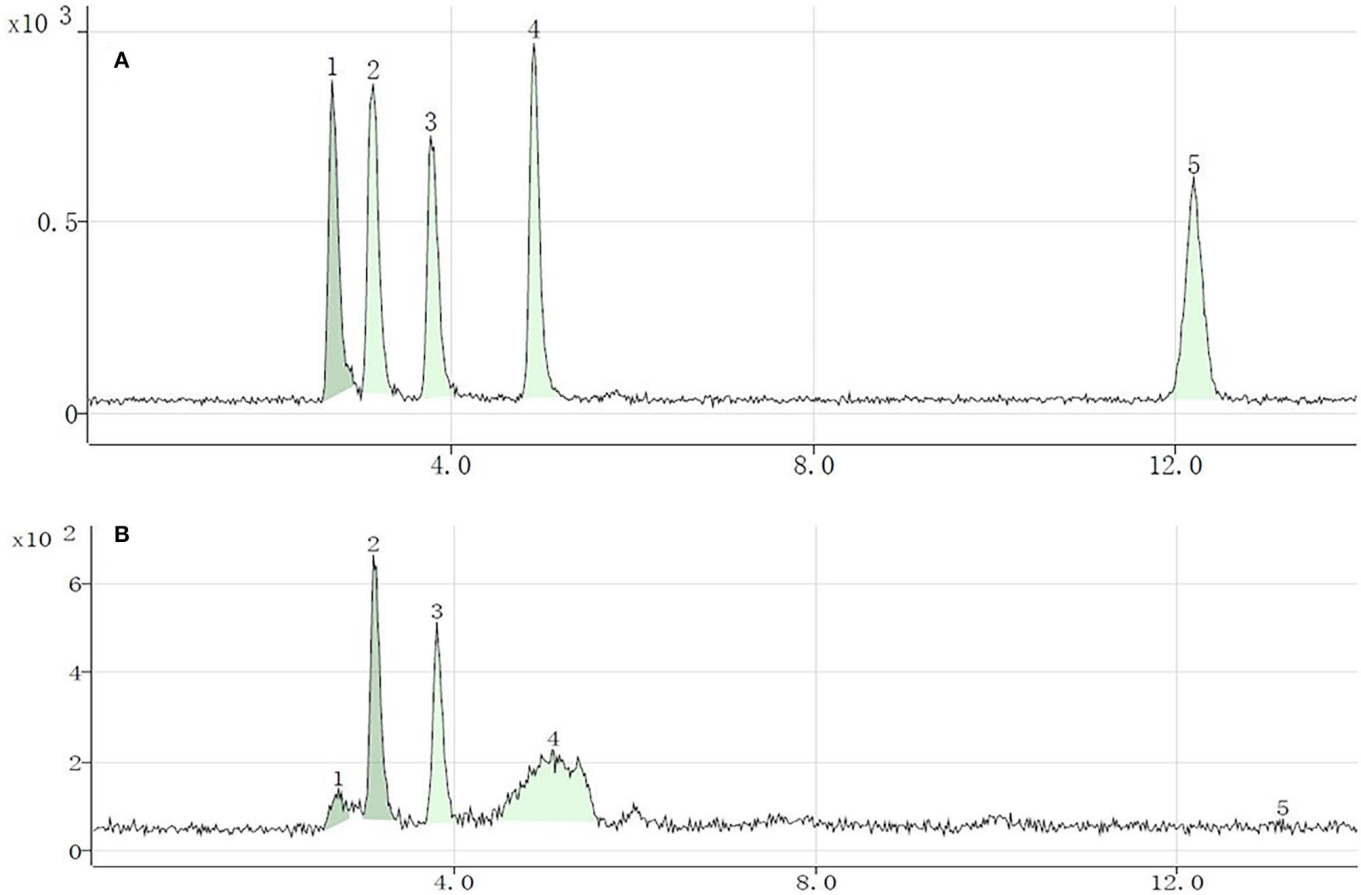
Chromatograms of different selenium (Se) species in standard solution (10 μg/L) **(A)** and Se-enriched germinated soybean sprouts **(B)**. Selenocystine (1), methylselenocysteine (2), selenomethionine (3), selenite (4), and selenate (5).

**Figure 2:**
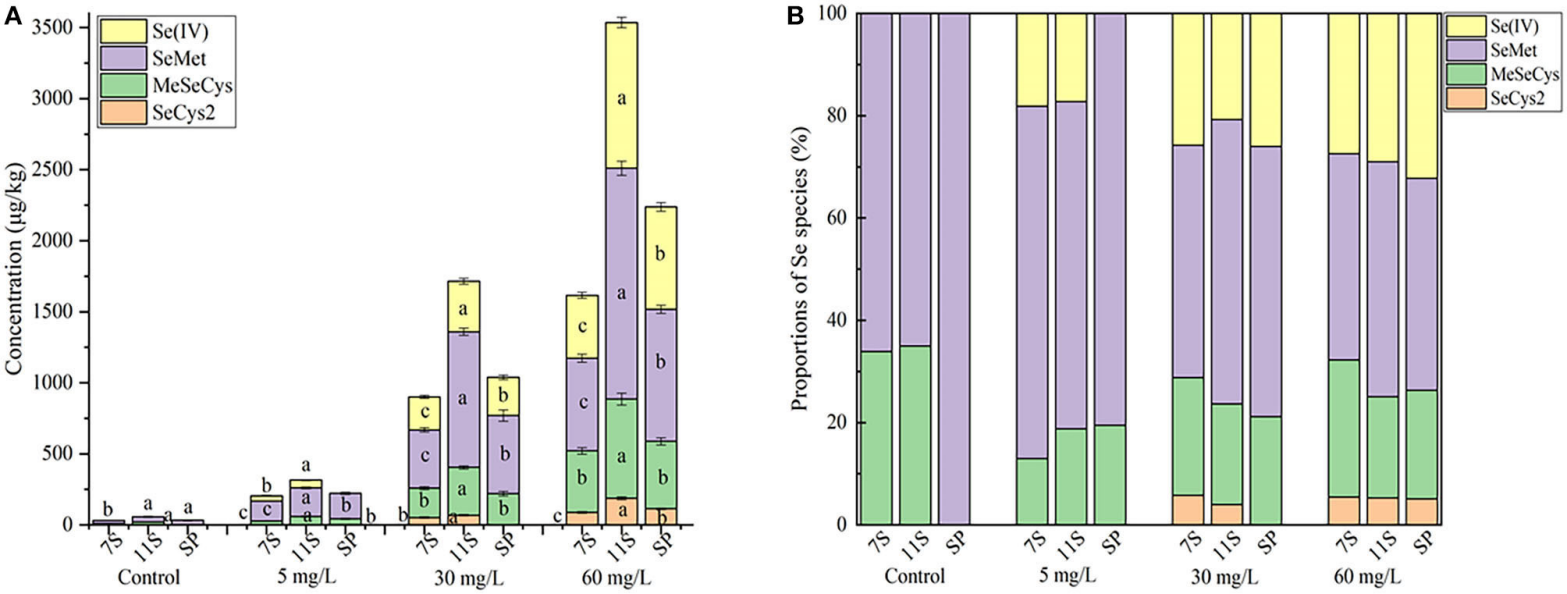
Concentration **(A)** and proportions **(B)** of Se species in soybean and proteins under different treatments. Different letters are used to show significant differences at various treatments (*p* < 0.05).

Five Se species were separated and identified by HPLC-inductively coupled plasma mass spectrometry; the chromatograms are shown in Figure 1 (A, standard solution; B, sample). The retention times of standard SeCys_2_, MeSeCys, SeMet, selenite, and selenate were 2.694, 3.146, 3.832, 4.937, and 13.019 min, respectively, and the recoveries were calculated through the sum of different Se species to total Se ranging from 77.7 to 97.5%. Of the five Se species, SeMet and MeSeCys were the dominant Se species (Figure 2), accounting for 41.5–80.5 and 19.5–21.2% in GS-Se with different concentrations of Se treatment, respectively. However, SeCys_2_ was only found in the germinated soybean with Se treatment above 30 mg/L, and selenate was not found in any of the treatments. The proportion of organic Se (sum of SeCys_2_, MeSeCys, and SeMet) decreased from 100 to 67.8%, as the Se treatment increased from 0 to 60 mg/L, and the proportion in 7S and 11S was higher than that in SP; however, the percentage of organic Se in 7S and 11S was 76.2 ± 4.9% and 77.7 ± 6.0%, respectively. There was no significant difference in the proportion of organic Se between the two protein fractions at the same level of Se treatment. Furthermore, the concentrations of different species were all higher in 11S than those in 7S by 31.5–150%.

### Changes in Amino Acid Content

The amino acid composition of soybean sprouts subjected to Se biofortification is listed in Table 2. The content of glutamic acid was the highest in all treatment groups (17.42–23.70%), followed by aspartic acid (10.71–12.41%), while the contents of cysteine (0.82–1.09%) and methionine were the lowest (0.72–1.45%). Isoleucine and leucine showed an increasing trend after germination, while most of the other amino acids increased slightly, without significant differences. The total amino acid content increased significantly after germination in both 7S and 11S. Se treatment had no significant effects on amino acids but decreased methionine in 11S. The amino acid content in 7S was significantly higher than that in 11S.

**Table 2:** Total amino acid concentration during soybean germination with different concentrations of Se treatment (g/100 g).

**Amino acids**	**7S-RS**	**7S-GS**	**7S-GS-Se30**	**11S-RS**	**11S-GS**	**11S-GS-Se30**
Aspartic (Asp)	9.01 ± 0.35 a	9.70 ± 0.41 a	9.23 ± 0.16 a	6.23 ± 0.18 a	6.52 ± 0.42 a	6.66 ± 0.28 a
Threonine (Thr)	2.46 ± 0.20 b	2.50 ± 0.16 b	2.52 ± 0.10 b	2.92 ± 0.14 a	2.95 ± 0.06 a	2.82 ± 0.20 ab
Serine (Ser)	3.36 ± 0.16 a	3.37 ± 0.08 a	3.34 ± 0.14 a	2.55 ± 0.04 b	2.44 ± 0.16 b	2.38 ± 0.07 b
Glutamate (Glu)	17.88 ± 0.83 a	17.99 ± 0.54 a	17.05 ± 0.34 a	11.90 ± 0.25 b	11.55 ± 0.33 bc	10.50 ± 0.31 c
Proline (Pro)	3.00 ± 0.10 a	3.05 ± 0.18 a	3.04 ± 0.16 a	1.91 ± 0.03 b	2.07 ± 0.17 b	2.21 ± 0.10 b
Glycine (Gly)	3.56 ± 0.13 c	3.56 ± 0.17 c	3.66 ± 0.14 bc	4.08 ± 0.13 a	4.01 ± 0.11 a	3.92 ± 0.08 ab
Alanine (Ala)	2.95 ± 0.06 a	2.97 ± 0.16 a	2.88 ± 0.20 a	2.72 ± 0.08 a	2.78 ± 0.07 a	2.89 ± 0.13 a
Cysteine (Cys)	0.65 ± 0.06 a	0.64 ± 0.06 a	0.55 ± 0.03 a	0.62 ± 0.01 a	0.65 ± 0.04 a	0.63 ± 0.03 a
Valine (Val)	2.98 ± 0.10 ab	3.14 ± 0.06 a	2.85 ± 0.06 b	2.90 ± 0.10 b	2.89 ± 0.01 b	2.45 ± 0.13 c
Methionine (Met)	0.54 ± 0.03 d	0.75 ± 0.04 bc	0.71 ± 0.01 bc	0.79 ± 0.04 b	0.88 ± 0.04 a	0.67 ± 0.03 c
Isoleucine (Ile)	2.51 ± 0.10 b	2.92 ± 0.11 a	2.67 ± 0.10 b	1.91 ± 0.10 d	2.27 ± 0.03 c	2.02 ± 0.11 d
Leucine (Leu)	7.00 ± 0.25 b	7.79 ± 0.25 a	7.79 ± 0.08 a	5.02 ± 0.18 d	6.03 ± 0.21 c	6.24 ± 0.17 c
Tyrosine (Tyr)	2.89 ± 0.24 ab	3.07 ± 0.17 a	3.00 ± 0.10 ab	2.48 ± 0.04 c	2.71 ± 0.13 abc	2.63 ± 0.14 bc
Phenylalanine (Phe)	3.32 ± 0.08 a	3.31 ± 0.13 a	3.12 ± 0.13 a	2.35 ± 0.18 b	2.48 ± 0.04 b	2.25 ± 0.11 b
Lysine (Lys)	4.11 ± 0.14 a	4.20 ± 0.11 a	4.02 ± 0.18 a	2.60 ± 0.16 b	2.77 ± 0.07 b	2.55 ± 0.11 b
Histidine (His)	2.64 ± 0.10 c	2.74 ± 0.16 c	2.55 ± 0.16 c	1.68 ± 0.14 d	4.02 ± 0.13 b	6.02 ± 0.21 a
Arginine (Arg)	6.58 ± 0.10 a	6.47 ± 0.31 a	5.98 ± 0.13 b	4.11 ± 0.11 c	3.83 ± 0.16 cd	3.42 ± 0.11 d
Total	75.44 ± 0.03 b	78.17 ± 1.20 a	74.96 ± 0.34 b	56.77 ± 1.24 d	60.85 ± 1.44 c	60.26 ± 0.47 c

*Different letters are used to show significant differences at various treatments (p < 0.05)*.

### Subunit Composition of Proteins

**Figure 3:**
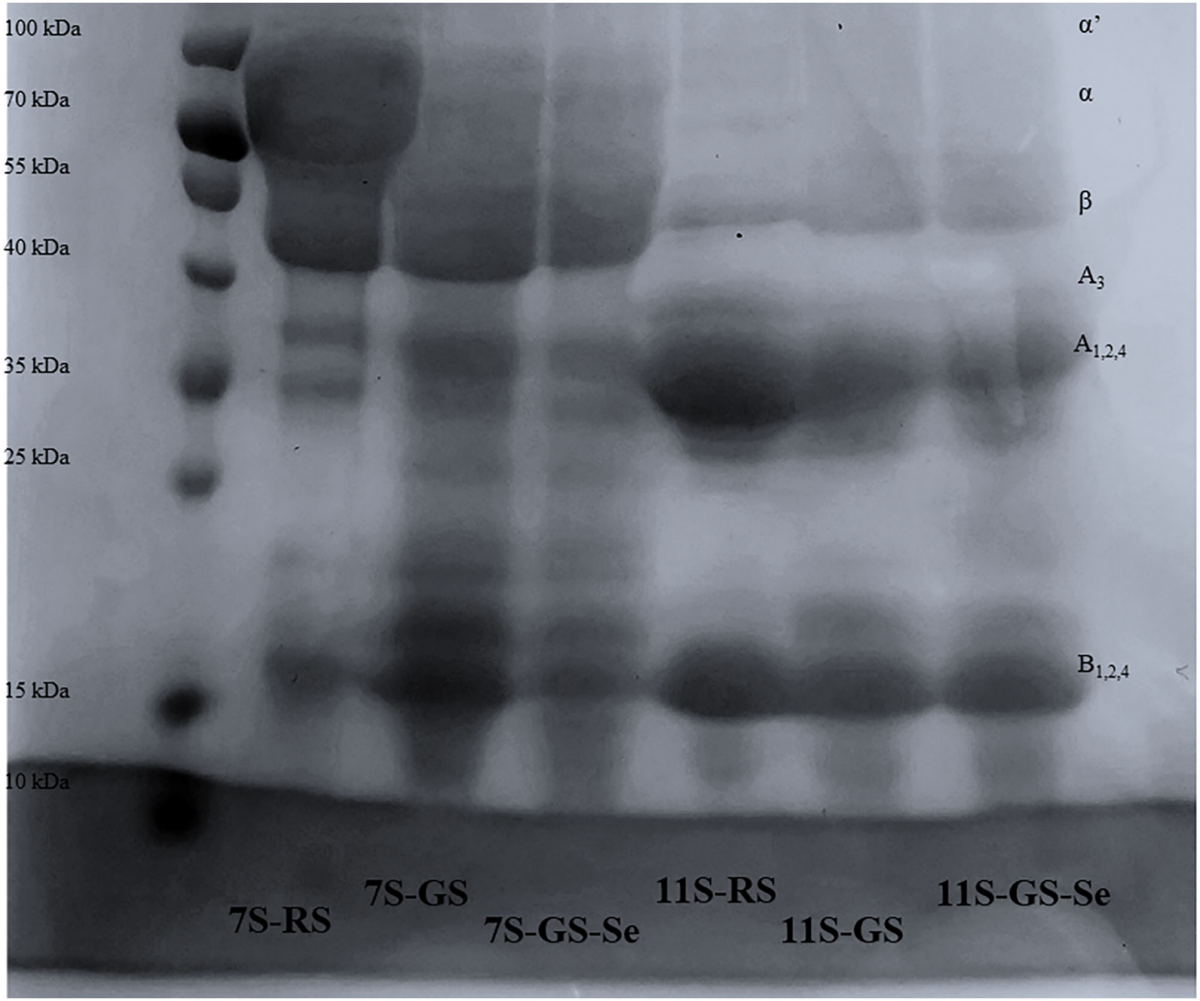
Sodium dodecyl sulfate-polyacrylamide gel electrophoresis patterns of different soybean proteins.

The subunits of different protein fractions (7S and 11S) from RS, GS, and GS-Se60 were analyzed by SDS-PAGE, and the results are shown in Figure 3. The molecular weight of 7S was mainly distributed at ~70, 40, 35, and 15 kDa, and the molecular weight of 11S was mainly distributed at ~37, 34, and 17 kDa. Both 7S and 11S shared a similar subunit composition in RS, GS, and GS-Se60, and there was no disappearance of the protein bands or appearance of new protein bands under Se treatment. Moreover, 7S and 11S were partially degraded into peptides with a low molecular weight due to germination, and the molecular weights of some proteins were lower than 15–25 kDa.

### Secondary Structural Composition of Different Soybean Proteins

The percentages of α-helix, β-sheet, β-turn, and random coil secondary structures of different protein fractions determined by FTIR spectroscopy in all the samples are shown in Table 3. The order of the secondary structure of proteins in all treatments was as follows: β-sheet, β-turn, random coil, and α-helix. Compared to those in RS, germination increased secondary structural components of different proteins in α-helix and β-turn and decreased them in β-sheet and random coil in 7S. Conversely, germination increased secondary structural components of proteins in random coil and decreased them in α-helix, β-sheet, and β-turn in 11S compared with those in RS. Comparing the structural composition percentages in the control and Se biofortification samples, the α-helix decreased with an increase in the Se concentration, while the other structural compositions were not significantly different, and the secondary structures of 11S showed no significant differences between the control and Se biofortification samples.

**Table 3:** Secondary structural compositions of different proteins (%).

**Protein**	**α-Helix**	**β-Sheet**	**β-Turn**	**Random coil**
7S-RS	15.26 ± 0.77 c	44.55 ± 3.37 a	20.35 ± 0.84 b	19.84 ± 3.31 a
7S-GS	21.14 ± 2.12 a	40.75 ± 3.54 ab	22.85 ± 1.08 a	19.42 ± 0.85 a
7S-GS-Se5	17.49 ± 0.56 bc	40.73 ± 0.01 ab	22.06 ± 0.09 ab	20.07 ± 0.02 a
7S- GS-Se30	19.02 ± 1.00 ab	39.02 ± 1.34 b	22.23 ± 0.92 ab	19.73 ± 0.60 a
7S- GS-Se60	16.64 ± 0.94 c	40.42 ± 1.62 ab	23.52 ± 2.16 a	19.42 ± 0.39 a
11S-RS	21.43 ± 0.51 a	43.68 ± 1.16 a	22.52 ± 1.82 b	12.36 ± 0.15 c
11S-GS	17.13 ± 0.67 b	39.51 ± 1.50 bc	21.73 ± 2.01 b	21.63 ± 0.17 a
11S- GS-Se5	18.09 ± 0.72 ab	38.71 ± 0.41 c	21.98 ± 0.67 b	21.22 ± 0.98 a
11S- GS-Se30	15.92 ± 1.07 b	41.35 ± 0.73 ab	23.55 ± 0.81 ab	19.42 ± 2.95 ab
11S- GS-Se60	16.10 ± 3.82 b	38.99 ± 0.60 bc	27.95 ± 2.72 a	16.96 ± 1.69 b

*Different letters are used to show significant differences at various treatments (p < 0.05)*.

### Functionality of Different Protein Fractions

**Figure 4:**
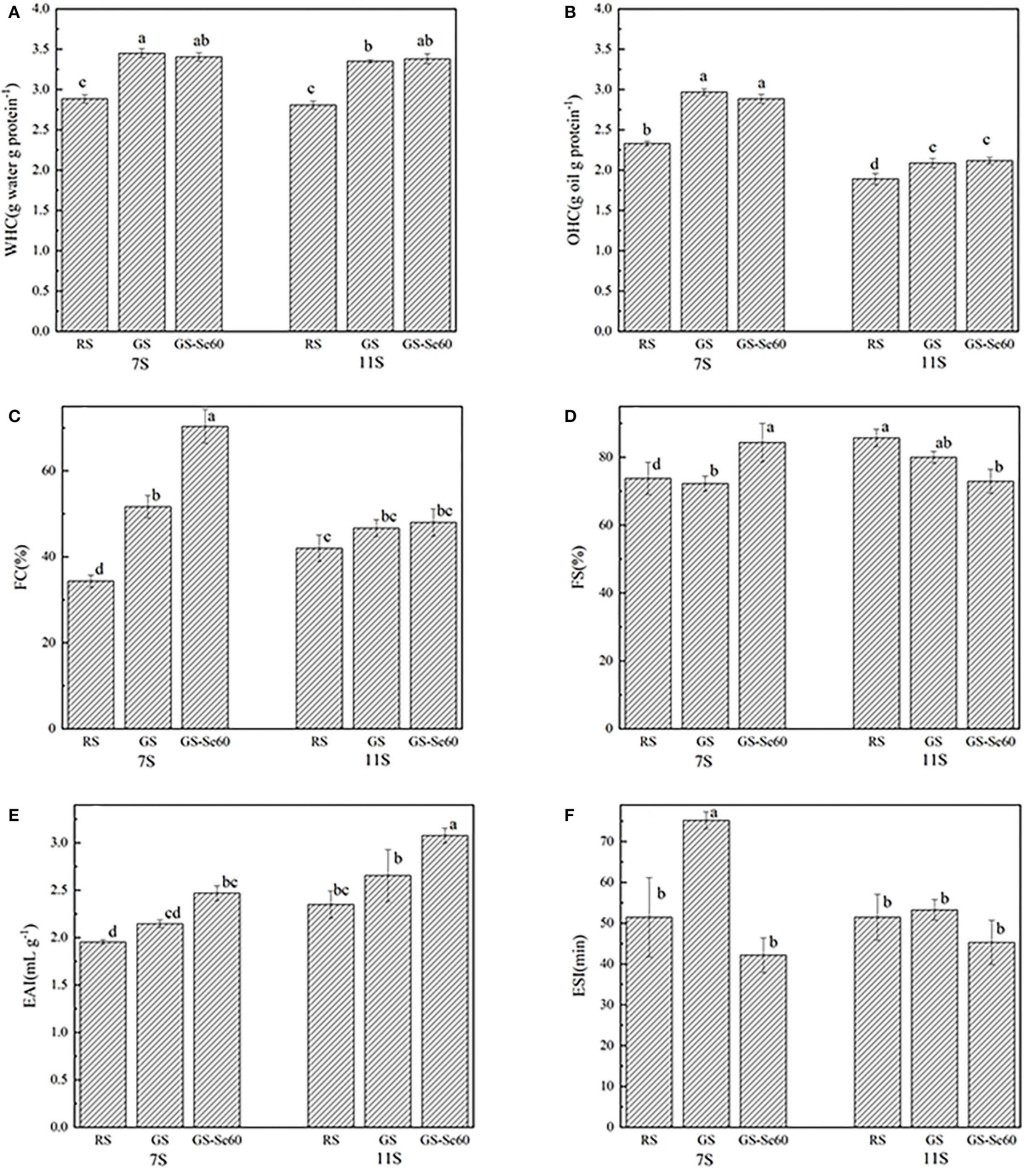
Functional properties of different soybean proteins treated with or without Se. **(A)** Water holding capacity (WHC), **(B)** oil holding capacity (OHC), **(C)** foaming capacity (FC), **(D)** foam stability (FS), **(E)** emulsion activity index (EAI), and **(F)** emulsifying stability index (ESI). Different letters are used to show significant differences at various treatments (*p* < 0.05).

The WHC and OHC of proteins from GS were significantly higher than those from RS, increasing by 18.17–20.28% in WHC and 10.40–27.32% in OHC, while Se treatment showed no significant effects on either WHC or OHC within the same treatment. The WHC of 7S and 11S from GS-Se60 was not significantly different; however, the OHC of 7S was significantly higher than that of 11S, increasing by 23.28–42.17% (Figures 4A,B). The FC of 7S increased with germination, and Se treatment promoted the FC of 7S (Figure 4C); however, there were no significant differences in 11S. Germination showed no significant effects on FS in both 7S and 11S (Figure 4D); the FS in 7S increased under Se treatment but showed no significant differences in 11S. The EAI of 7S and 11S decreased after germination, whereas Se biofortification had no significant effects on either 7S or 11S (Figure 4E). The ESI increased significantly under Se biofortification, whereas Se had no significant effects on 11S (Figure 4F).

### Antioxidant Activities

**Figure 5:**
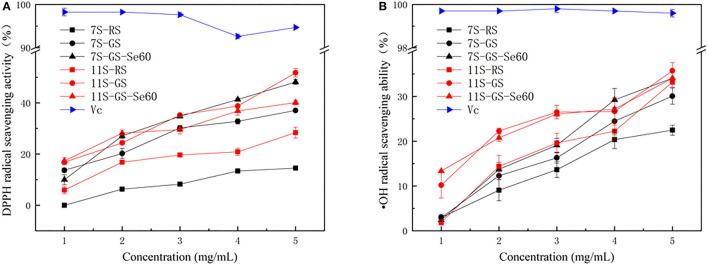
1,1-Diphenyl-2-picrylhydrazyl (DPPH) radical scavenging activity **(A)** and hydroxyl free (·OH) radical scavenging ability **(B)**.

Germination promoted the ability of soybean protein to scavenge DPPH free radicals and hydroxyl radicals in both 7S and 11S, and the DPPH and hydroxyl radical-scavenging activities of the samples increased in a concentration-dependent manner (Figure 5). Se improved the ability of soybean protein to scavenge DPPH free radicals and hydroxyl radicals in 7S; however, this ability decreased in 11S. The order of DPPH scavenging ability among different proteins at 5 mg/ml was as follows: 11S-GS (51.8 ± 1.69), 7S-GS-Se60 (48.1 ± 0.99), 11S- GS-Se60 (40.1 ± 0.94), 7S-GS (37.0 ± 0.49), 11S-RS (28.3 ± 2.16), and 7S-RS (14.5 ± 0.85). The order of hydroxyl radical-scavenging ability among different proteins at 5 mg/ml was as follows: 11S-GS (35.74 ± 1.81), 7S-GS-Se60 (34.1 ± 1.40), 11S-GS-Se60 (34.08 ± 1.40), 11S-RS (33.18 ± 1.13), 7S-GS (30.1 ± 1.40), and 7S-RS (22.5 ± 1.13).

## Discussion

### Total and Species Se Distribution in Different Proteins

The results indicated that germination in the presence of a selenite solution is an efficient method for Se biofortification of germinated soybeans, similar to a previous study (36) in which the Se concentration in soybean increased from 4.6 to 10,100 μg/kg. Se is beneficial at low levels but toxic at high levels, and the margin between deficiency and excess is narrow (13). To achieve health benefits, it has been suggested that Se intake should exceed the adult recommended dietary allowance of 50–60 μg/day and stay below the tolerable upper intake level of 400 μg/day (37). In our study, the concentration of total Se in GS-Se60 was 2,822 μg/kg, which was 79.8 times higher than that in RS. The ability of 11S to enrich Se was markedly higher than that of 7S in all treatment groups during germination. To the best of our knowledge, there has been no study investigating Se distribution in different germinated soybean proteins, and the results were consistent with those of a study on soybeans cultivated on farms. Deng et al. (7) found that the concentrations of Se in 11S are significantly higher (38.6%) than those in 7S; Wang et al. (38) also found that the concentration of Se in 11S is considerably higher (12.1%) than that in 7S in natural Se-enriched raw soybeans in Enshi, indicating that the 11S can bind Se more efficiently than 7S. However, Zhao et al. found that the Se content of 7S and 11S from Se biofortification soybeans is about 9.9-fold higher compared with ordinary soybeans, and there is no significant difference between 7S and 11S (25), which may be due to differences in the methods of soybean cultivation, extraction, and analysis. Above all, the ability of 11S to enrich Se in raw soybeans compared that of 7S was not greater than that of Se-enriched soybeans during germination, indicating that it is much easier to combine Se with 11S than with 7S during germination.

SeMet and MeSeCys were the dominant Se species in the germinated soybeans, indicating that selenite is easily converted to organic species during germination and can be efficiently incorporated into proteins (39). The decreased proportion of organic Se with increasing Se treatment concentrations was in line with a previous study, in which the proportion of organic Se in plants decreased from 88 to 80%, indicating that an increased concentration of Se in fertilizer will reduce the efficiency of conversion from inorganic to organic Se (30). Similar to the results for the total Se contents, the concentrations of different species were far higher in 11S than those in 7S. A plausible reason for this phenomenon is that S-containing amino acids in 7S were lower than those in 11S, and a previous study found that Se is incorporated into proteins mainly through taking the place of S in the S-containing amino acids (7). Previous studies also found that Se is mainly incorporated into proteins with low-molecular-weight compounds, such as in selenium-enriched mushrooms (not more than 16 kDa), soybean (15–20 kDa), Se-enriched rice (<36.3 kDa), and Se-enriched *Tenebrio molitor* larvae (<40 kDa) (11, 40–42). The contents of low-molecular-weight subunits in 11S were higher than those in 7S, and this may also be the reason for higher Se content in 11S.

### Effects of Se on Amino Acids and Protein Structure

Germination had no significant effects on most of the amino acids, with amino acid contents increasing slightly without significant differences; however, germination led to a significant increase in the total amino acids. Yang et al. found that the germination process leads to a significant decrease in some amino acids (23). However, Gao et al. found that germination increased the amino acid concentration of soybeans (22). The differences between the previous results and our work could be due to differences in the varieties of soybean and conditions during germination. Se treatment had no significant effect on amino acids but decreased methionine in 11S. A similar phenomenon has also been reported by Zhao et al. (25), who found that Se had no significant effect on the concentrations of most amino acids in raw soybean and only caused a reduction in concentrations of cysteine and methionine. This result could be due to Se taking the place of S in these two amino acids so that parts of methionine and cysteine are converted to SeMet and SeCys (43).

The subunits of 7S and 11S extracted from RS, GS, and GS-Se samples were analyzed by SDS-PAGE, and there were no new bands formed and existing bands did not disappear, indicating that Se biofortification did not make protein subunits degrade or aggregate. However, 7S and 11S were partially degraded into low-molecular-weight peptides due to germination, which is consistent with previous research (23, 44). Previous studies have found that 7S and 11S share the same bands between Se-enriched and ordinary raw soybeans (7, 11, 25). These studies indicate that the Se incorporated into proteins does not significantly affect the subunit composition of soybean proteins. However, Luo et al. (30) found that as Se fertilizer increases to 100 g/ha, the protein subunit of molecular weight of ~30 kDa moves upward, because the high Se content leads to protein subunit binding. The different phenomena observed in these studies may be due to the concentration of Se. A low concentration of Se does not change the protein subunit distribution, and a high concentration of Se can influence the molecular weight of proteins.

Based on the results detailed in Table 3, it can be concluded that Se influenced the secondary structures of proteins to some extent. Se is bound to amino acids and then incorporated into soybean proteins, which may influence the protein structure and also the secondary structure (17). It has been speculated that the decrease in the concentration of Cys and Met is caused by the replacement of S with Se in these two amino acids, both being hydrophobic (30). If Se converts S–S into Se–Se, indicating that the disulphide bond has changed, together with the atomic size and ionization of Se, then the secondary structure will be influenced (45). Zhao et al. (25) speculated that Se could influence the secondary structures of 7S and 11S in raw soybeans. However, Deng et al. (7) found a different phenomenon that Se has no significant effects on the secondary structure of 7S and 11S in raw soybean. It is possible that the interference of Se species with the contents of sulfhydryl groups and disulfide bonds is negligible because the Se content in the protein is much lower than that of S (30). Therefore, the specific reasons for the effect of Se on protein secondary structure need to be further investigated.

### Effects of Se on Different Protein Functional Properties

The improvement in protein WHC of 7S and 11S by germination was consistent with the results of SDS-PAGE experiments, in which 7S and 11S are hydrolysed by enzymes during germination (44), and the high WHC of proteins could be attributed to the high hydrophilicity of the soybean proteins (3). It seems that with the increased grade of hydrolysis, the protein may easily hold more water, and there is a higher WHC. The present study found that Se had no significant effect on the WHC and OHC, which is consistent with the study conducted by Deng et al. (7). However, Lazo-Velez et al. found that germinated soybeans enriched with Se promote WHC (46). The water-holding ability is an important functional property in food processing, such as in the preparation of sausages, pumped meats, and confections, as it increases product yields (47). Higher oil absorption can be useful for ingredients in meat, sausages, and dairy products, where the oil-holding ability affects the texture of the food (48).

Germination increased the FC of 7S and 11S and decreased the FS, which is consistent with previous studies (26, 49). This may be because the increased concentration of polypeptide, which is produced during soybean germination, could promote FC by incorporating more air (23). According to a previous study, a reduction in FS may be due to the low strength of micropeptides that maintain the stability of the foam (49). Therefore, the increased FC and decreased FS of GS in this study could be caused by the high solubility and low molecular weight of peptides. These low-molecular-weight peptides do not promote stable foams because of the reduced interactions between proteins (26, 50). The low FS is caused by weak interfacial surface films between bubbles, and the bubbles tend to collapse (51). The FC and FS of protein are important for processing cakes, whipped desserts, and ice cream (47). Interestingly, the 7S of GS-Se had both high FA and FS, and studies have shown that the FS depends on the rheological properties of the protein-membrane, as well as the protein-protein interactions and environmental factors (52). Thus, this result may be due to Se producing greater electrostatic repulsion, thus stabilizing the foams.

Previous studies have reported that the composition and structure of protein would influence emulsifying properties (53). In the present study, germination increased the EAI and ESI of 7S and 11S, and Se treatment increased EAI but decreased ESI. A similar phenomenon in germination was found in some previous studies, in which EAI and ESI were influenced by germination time (22, 44). Yang et al. found that germination can significantly increase the EAI of soybean protein but has no effect on ESI (23). Enzymatic hydrolysis could promote EAI and ESI by increasing the solubility of protein, which is important for emulsifying properties (26). A high solubility allows proteins to rapidly diffuse and adsorb at the interface between water and oil (3). Germination improved the EAI and ESI compared with those of raw soybean, which may also be caused by the increased solubility, so proteins can migrate to the interface rapidly (54). Overall, the changes in the protein content, amino acid composition, and protein structure might affect the functional properties of soybean proteins (17).

### Effects of Germination and Se on the Antioxidant Capacity

Previous studies have shown that germination can improve the antioxidant capacity of many products, such as soybean (55), kale, kohlrabi (56), and wheat (57), but these studies have mainly focused on the whole product powder; they attributed the improved antioxidant capacity to phenolics and flavonoids. This has also been verified by other studies, where higher antioxidant capacities are observed in germinated soybean mainly because of an increase in the concentration of total isoflavones (58, 59). Gao et al. found that soybean sprout protein effectively eliminates DPPH and •OH free radicals, showing that germination can strengthen the antioxidant activity of soy proteins (22). In the present study, we further investigated 7S and 11S, the two main protein fractions, and the antioxidant capacities of both proteins were improved through germination; the plausible reason is that germination can transform protein into small peptides, which have strong radical-scavenging abilities (60).

Selenium improved the ability of 7S to scavenge DPPH-free radicals and hydroxyl radicals in our study, which has also been found in previous studies (12, 61, 62); Se-containing proteins exhibit significantly higher antioxidant ability *in vitro* than proteins without Se (63). Se can promote both enzymatic and non-enzymatic antioxidant systems, such as GPx and glutathione (64). Se also increases some hydrophobic amino acid content in proteins, thereby enhancing the antioxidant capacity (40). A diselenide bridge, formed during the oxidation of two neighboring Cys residues, is longer than a disulfide bridge and has a lower redox potential (65). Therefore, a plausible reason is that 7S-containing Se–Se bonds tend to have higher antioxidant capacity than that containing S-S bonds.

However, Se had opposite effects on the antioxidant activities of 11S in our study, which cannot be reasonably explained, as the antioxidant mechanism of Se-enriched soy protein may be related to one or more of these mechanisms. Antioxidant properties are promoted with an increased concentration of Se in proteins (11). The antioxidant ability is affected not only by Se content and species but also by other factors, such as protein and amino acids (66), or by reducing sugars, ascorbic acid, and organic acids, among others, which may influence the evaluation of the antioxidant ability (64). Because the antioxidant activity is influenced by so many factors, the specific mechanism by which Se enhances the antioxidant activity of the protein is still uncertain, and the changes in antioxidant activity need to be further studied to clarify how Se affects the antioxidant activity. In addition, *in vitro* experiments are widely used to evaluate antioxidant capacity. However, these reactions are not just involved in the antioxidant enzymes in organisms, and evaluation methods should simulate real physiological conditions in organisms or involve tests using animal models.

## Conclusion

SeMet and MeSeCys were the main Se species in the germinated soybeans, and dominant proteins 7S and 11S had different Se enrichment abilities. Se treatment had no significant effects on amino acids but decreased methionine in 11S. Moreover, the contents of α-helix decreased with increasing Se concentration, while the other structures were not significantly different. Se treatment had no significant effects on WHC and OHC but increased the FC and EAI of 7S, but only the EAI of 11S. Furthermore, Se treatment increased the antioxidant capacity in 7S but had no significant effects on that of 11S. The present study provides initial insight into the Se distribution in different germinated soybean dominant proteins and its effects on protein structure, functional properties, and antioxidant activity. The results provide important evidence for the development of efficient natural Se-enriched food supplements and the processing of Se-enriched germinated soybean protein.

## Data Availability Statement

The original contributions presented in the study are included in the article/Supplementary Material, further inquiries can be directed to the corresponding authors.

## Author Contributions

YH contributed to methodology, study design, and manuscript writing. NL, YX, and YL investigated the study. BF and LT contributed to data analysis. FW, CB, and PM contributed to study design, manuscript review, and funding acquisition. All authors have read and agreed to the published version of the manuscript.

## Funding

This study was supported by the Special National Key Research and Development Plan (2021YFD1600101), the Quality and Safety of Agricultural Products (Risk Assessment) from the Chinese Ministry of Agriculture and Rural Affairs (Grant No. GJFP2019001), and the China Agriculture Research System (CARS-04).

## Conflict of Interest

The authors declare that the research was conducted in the absence of any commercial or financial relationships that could be construed as a potential conflict of interest.

## Publisher's Note

All claims expressed in this article are solely those of the authors and do not necessarily represent those of their affiliated organizations, or those of the publisher, the editors and the reviewers. Any product that may be evaluated in this article, or claim that may be made by its manufacturer, is not guaranteed or endorsed by the publisher.
