# Supplementary material for: Selenium Biofortification of Soybean Sprouts: Effects of Selenium Enrichment on Proteins, Protein Structure, and Functional Properties
Source: Front Nutr. 2022 May 3;9:849928. doi: 10.3389/fnut.2022.849928 (PMC9113265; doi:10.3389/fnut.2022.849928)
Supplement: Supplementary file 1 [file Table_1.DOCX]

Supplementary Material

**Preparation of 7S and 11S:** The fat parts were removed from the soybean powder, then 7S and 11S were extracted from the defatted soybean powders. About 100 g soybean powder was added to beaker (5 L), and 3 L petroleum ether was added. Then removed petroleum ether after 3 h stirring and extraction, and the remaining solid was collected, air-dried, and stored at 4 °C. About 60 g the powder sample was added with a phosphate buffer solution (2000 mL, pH=8) and extracted at 45 ℃ for 45 min, the supernatant was collected and adjusted to pH 6.4 with HCl solution after centrifugation, then solid NaCl was added to a concentration of 30 mol L^-1^. Then the supernatant was centrifuged at 5000 × g for 10 min after 12 h at 4 ℃, and precipitate was collected as 11S. The remaining supernatant was adjusted to pH 5.25 using 1 mol L^–1^ HCl and centrifuged. The precipitate was discarded and the pH of the supernatant was adjusted to 4.8 using 1 mol L^–1^ HCl. The supernatant was centrifuged and the resulting precipitate was collected as 7S.

**Amino acids analysis:** About 0.3 g protein sample was added into a hydrolysis tube with 10 mL HCl solution (6 mol L^-1^), then the tube was filled with nitrogen for protection and tightened up. After been kept at 110 ℃ for 22 h, the hydrolysate was filtered into a 50-mL volumetric flask after cooled to room temperature. The final volume of the filtrate was adjusted to 50 mL with water and shake well. The filtrate (exactly 1.0 mL) was pipetted into a 15-mL flask, then the sample was passed through a 0.22-μm filter membrane. 17 amino acids of the samples were determined by automatic amino acid analyzer (LA8080; Hitachi, Tokyo, Japan).
